# Placental Growth Factor in Diabetic Retinopathy: Disease-Selective Signaling and Mechanistic Rationale for Targeted Therapy

**DOI:** 10.3390/ijms27167354

**Published:** 2026-08-17

**Authors:** Jongmin Kim, Won-Kyu Ju, Jae Yon Won

**Affiliations:** 1 POSTECH-Catholic Biomedical Engineering Institute, The Catholic University of Korea, Seoul 06591, Republic of Korea; 2 Viterbi Family Department of Ophthalmology, Viterbi Family Vision Research Center, Shiley Eye Institute, University of California San Diego, La Jolla, CA 92039, USA; wju@health.ucsd.edu; 3 Institute of Engineering in Medicine, Shu Chien—Gene Lay Department of Bioengineering, University of California San Diego, La Jolla, CA 92039, USA; 4 Department of Ophthalmology and Visual Science, Eunpyeong St. Mary’s Hospital, The Catholic University of Korea, Seoul 03312, Republic of Korea; 5 Catholic Institute for Visual Science, College of Medicine, The Catholic University of Korea, Seoul 06591, Republic of Korea

**Keywords:** diabetic retinopathy, placental growth factor, VEGFR1, VEGF-A, retinal angiogenesis, vascular leakage, inflammation

## Abstract

Chronic hyperglycemia disrupts the retinal neurovascular unit, making diabetic retinopathy (DR) the leading cause of adult vision loss. The hallmarks of DR include pathological angiogenesis, vascular leakage, and neurodegeneration. Although vascular endothelial growth factor A (VEGF-A) is a key mediator of these abnormalities and anti-VEGF therapies remain the standard treatment, many patients exhibit incomplete responses, and these therapies do not adequately address inflammation or fibrosis. Placental growth factor (PlGF), a VEGF family member that selectively binds to VEGFR1, has emerged as an important contributor to retinal neovascularization, vascular dysfunction, barrier breakdown, and microinflammation in DR. Unlike VEGF-A, PlGF modulates distinct responses among retinal neurons, glial cells, and vascular cells, with its abnormal expression closely associated with disease progression from non-proliferative to proliferative DR. This review summarizes the context-dependent functions of PlGF in the diabetic retina, compares PlGF–VEGFR1 signaling with VEGF-A–VEGFR2 pathways, and discusses preclinical and clinical evidence supporting PlGF inhibition as a potential complementary approach to existing anti-VEGF therapy.

## 1. Introduction

The retinal circulation is highly specialized and particularly susceptible to the metabolic and hemodynamic stress associated with diabetes [1]. Chronic hyperglycemia induces oxidative stress, low-grade inflammation, and ischemic changes, collectively shifting the retinal environment toward increased expression of pro-angiogenic and permeability-promoting factors [1,2,3]. Among these factors, vascular endothelial growth factor A (VEGF-A) is the most extensively studied. Through VEGFR2 activation, it promotes pathological angiogenesis and vascular permeability, and VEGF-A-targeted therapy has substantially improved outcomes for many patients with vision-threatening diabetic retinopathy (DR) and diabetic macular edema [1,3,4,5,6]. However, heterogeneous treatment responses, recurrent disease activity, and concerns regarding prolonged disruption of physiological VEGF signaling highlight the need for additional or more selective therapeutic targets [1,4,5,6].

**Figure 1:**
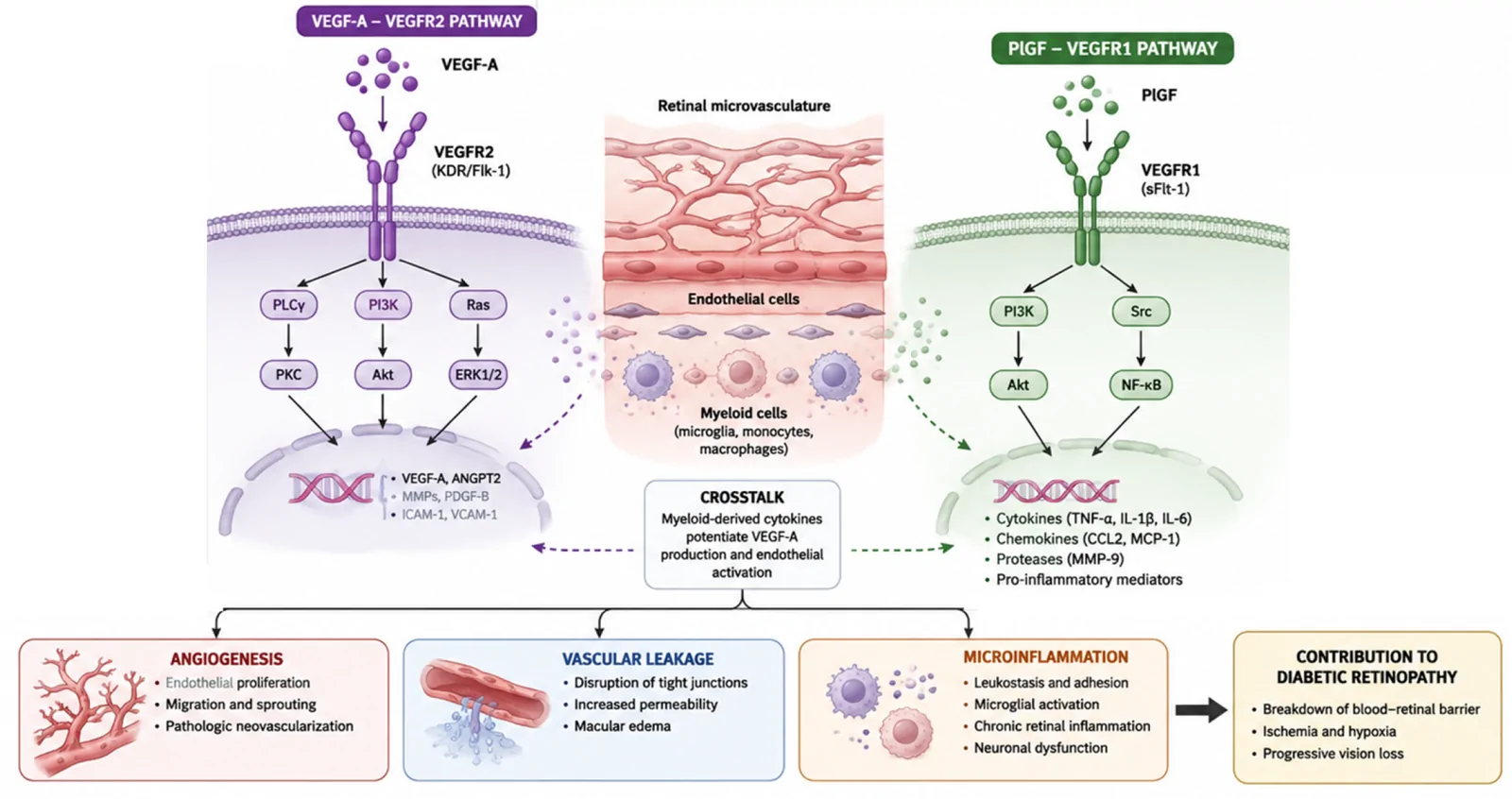
Overview of VEGF-A and PlGF signaling pathways in diabetic retinopathy. Schematic of VEGF-A–VEGFR2 and PlGF–VEGFR1 signaling pathways in retinal endothelial and myeloid cells and their roles in angiogenesis, vascular leakage, and microinflammation [4,7,12,13].

Placental growth factor (PlGF) has emerged as a potential therapeutic target in this context [7,8,9,10,11]. Unlike VEGF-A, which binds to both VEGFR1 and VEGFR2 and is essential for normal vascular development and homeostasis, PlGF primarily interacts with VEGFR1 and appears to have limited physiological importance but becomes selectively upregulated under pathological conditions [3,7,8,12,13]. In this review, “disease-selective signaling” refers to the context-dependent activation of the PlGF–VEGFR1 pathway in which PlGF preferentially promotes pathological angiogenesis, vascular leakage, inflammation, and tissue remodeling while exerting limited effects on physiological vascular homeostasis. This signaling profile makes PlGF an attractive therapeutic target because modulating the PlGF–VEGFR1 pathway may inhibit pathological retinal changes while preserving VEGF-A-mediated physiological functions. In diabetic and ischemic retinas, PlGF expression is upregulated and contributes to neovascularization, vascular leakage, and the recruitment and activation of microglia and macrophages [4,11,12,13]. These findings suggest that PlGF–VEGFR1 signaling is a disease-biased pathway that can be targeted to suppress pathological changes in DR while maintaining relatively intact physiological VEGF-A–VEGFR2 signaling [4,7,10,12,13] (Figure 1). Since the publication of earlier comprehensive reviews of PlGF biology in retinal vascular disease, including the influential review by Van Bergen et al., the field has expanded beyond the predominantly angiogenic and vascular perspective of PlGF signaling [3]. More recent studies have provided additional insight into VEGFR1-dependent signaling, metabolic and redox regulation, neuroinflammation and neurodegeneration, fibrotic remodeling, and the potential use of PlGF as a biomarker for disease stratification and therapeutic response. At the same time, the therapeutic landscape of diabetic retinopathy has evolved with the introduction of dual-pathway inhibition, higher-dose anti-VEGF therapy, and sustained-delivery strategies, alongside increasing interest in biomarker-guided treatment and precision ophthalmology. Against this background, the present review is intended not simply to provide an updated summary of the PlGF literature, but to offer an integrated conceptual framework linking vascular dysfunction, inflammation, metabolic stress, fibrosis, and neurodegeneration through PlGF–VEGFR1 signaling specifically for diabetic retinopathy. By framing these interconnected processes within the concept of disease-selective signaling, this review aims to clarify the mechanistic rationale, current limitations, and potential therapeutic positioning of PlGF-directed strategies within an increasingly individualized treatment landscape.

## 2. Context-Dependent Roles of PlGF in the Retina

In non-diabetic eyes, PlGF expression is low, and PlGF-deficient mice develop normal retinal vasculature, suggesting that this ligand is not essential for baseline vascular development or maintenance [1,4,13]. However, under diabetic or hypoxic conditions, PlGF expression increases in several retinal cell types, including endothelial cells, Müller glia, and mononuclear phagocytes [3,7,8,12,13].

Gain and loss-of-function studies support a causal role for PlGF in early microvascular abnormalities [4,12,14]. Sustained intraocular PlGF overexpression in rat eyes induces capillary tortuosity dilation, microaneurysm formation, and disruption of retinal pigment epithelial junctions, resembling early DR changes [14]. Conversely, genetic deletion or antibody-mediated inhibition of PlGF in diabetic mice reduces capillary dropout, pericyte loss, and neuronal damage while preserving the inner blood–retinal barrier [3,4,12,14]. These findings establish PlGF as a context-dependent mediator that remains quiescent under physiological conditions but promotes pathology in metabolically stressed and ischemic diabetic retinas [4,7,8,12,14].

## 3. Distinct and Overlapping Features of VEGF-A and PlGF Signaling

VEGF-A and PlGF belong to the same ligand family but differ in receptor utilization and functional profiles, with important implications for therapeutic development [7,8,10,12,13] (Table 1). PlGF may also indirectly modify VEGF-A signaling. Competition for VEGFR1 and the formation of VEGF/PlGF heterodimers can alter VEGF-A distribution between VEGFR1 and VEGFR2, thereby influencing downstream responses. This complexity may explain why dual-targeting agents such as aflibercept, which sequesters both VEGF A and PlGF, can exhibit clinical effects that differ from those of VEGF-A-specific inhibitors.

## 4. VEGF-A/VEGFR2 and PlGF/VEGFR1 Pathways in DR

In ischemia-driven diabetes, HIF-1α activation increases VEGF-A expression in retinal pigment epithelium, Müller glia, and endothelial cells, promoting VEGFR2-mediated angiogenesis, vascular permeability, and leukocyte adhesion [1,5,6,8]. VEGFR1 is also expressed on retinal endothelial cells, pericytes, microglia, and infiltrating macrophages, where PLGF activation stimulates ERK and NF-kB signaling [15,16]. These pathways enhance endothelial sprouting and promote the production of pro-angiogenic and pro-inflammatory mediators—including TNF-α, IL-1β, and chemokines—by myeloid cells. Direct VEGFR1 signaling also influences vascular permeability and induces inflammatory and angiogenic mediator production by macrophages and microglia [10,12,13].

PlGF is a central component of the VEGFR1 network, acting on endothelial cells to disrupt junction integrity and on myeloid cells to amplify inflammatory and pro-angiogenic responses, thereby linking metabolic stress and hypoxia to the vascular-inflammatory phenotype of DR [4,7,11,12,13]. In addition, pericyte–endothelial interactions, which maintain capillary stability, are disrupted in DR, and VEGFR signaling contributes into this cellular cross-talk [3,17,18]. Loss of these interactions promotes pericyte depletion, microaneurysm formation, and eventual neovascularization [17,18].

### 4.1. Structural and Signaling Basis for PlGF Selectivity

PLGF was initially characterized as a VEGF-family ligand with a more restricted signaling profile than VEGF-A because it binds predominantly to VEGFR1 and engages neuropilin-1 only under specific isoform-dependent conditions. In the retina, this selective receptor usage is important because VEGFR1 is expressed on endothelial cells, pericytes, and innate immune cells, enabling PlGF to regulate vascular and inflammatory responses in a disease-biased manner rather than acting as a general angiogenic driver. Experimental studies have shown that PlGF promotes endothelial migration, myeloid-cell activation, and inflammatory amplification while modulating VEGF-A availability through receptor competition and receptor cross-talk. These features support the view that PlGF is not merely a redundant VEGF-family member, but a context-dependent signal that becomes especially relevant in ischemic and diabetic tissues.

### 4.2. PlGF in Retinal Vascular Pathology

In DR, PlGF expression increases alongside hypoxia, oxidative stress, and inflammation. Clinical studies consistently demonstrate elevated PlGF levels in aqueous humor, vitreous fluid, and retinal tissues of patients with DR, particularly those with proliferative DR and diabetic macular edema, where levels correlate with disease severity and retinal ischemia. These findings support the translational relevance of experimental evidence and indicate that PlGF is closely associated with disease progression in DR [3,4,14,19,20,21,22,23].

### 4.3. Barrier Failure, Inflammation, and Metabolic and Redox Stress

**Figure 2:**
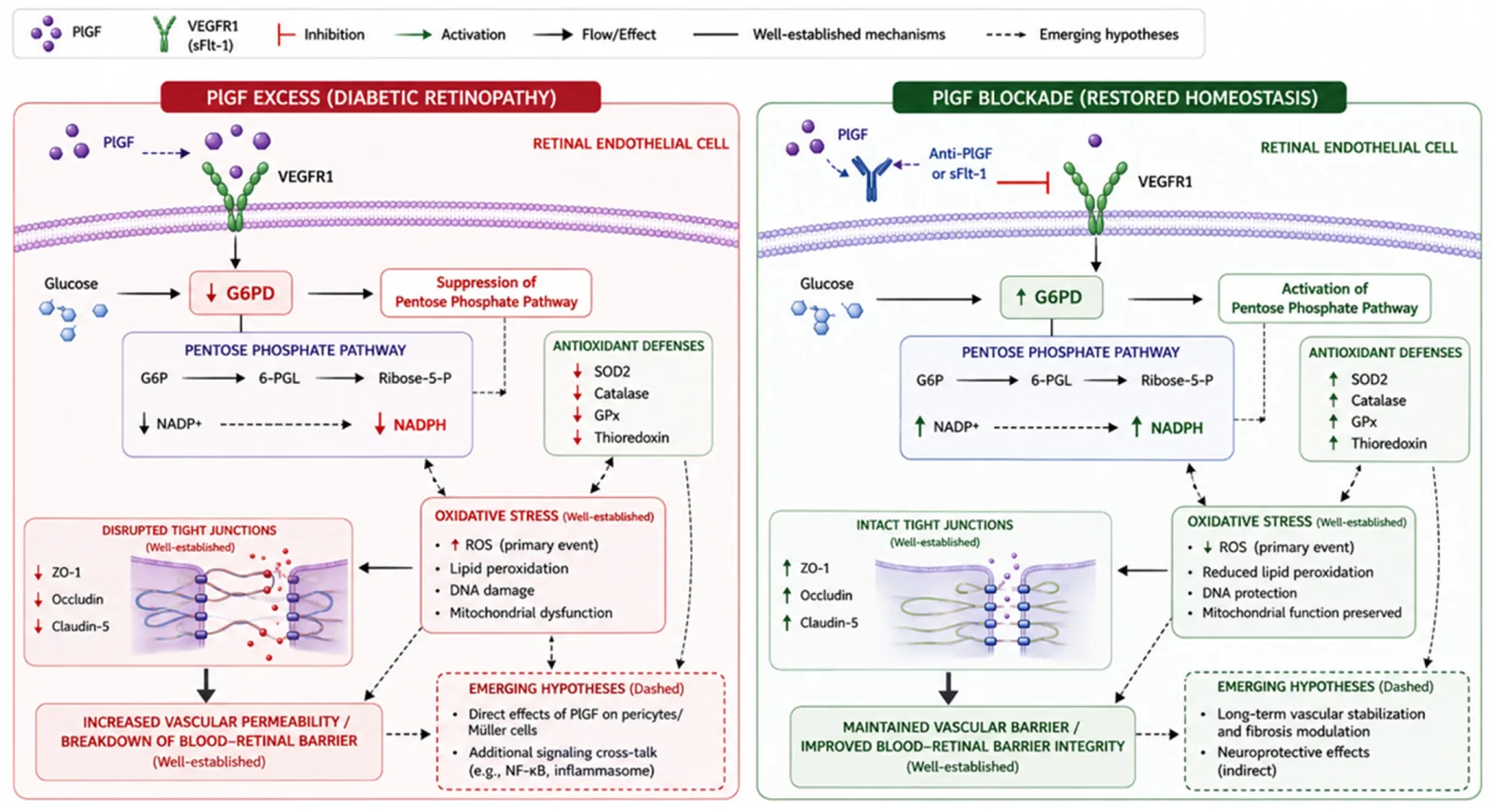
PlGF-dependent regulation of the PPP and antioxidant defenses. Schematic illustrating how PlGF suppresses G6PD and antioxidant proteins, increases ROS production, and disrupts tight-junction integrity, whereas PlGF blockade restores PPP flux and barrier stability [11,12,13,24].

A major consequence of PlGF–VEGFR1 signaling is disruption of the endothelial barrier. Studies in human retinal endothelial cells show that PlGF reduces trans-endothelial electrical resistance and disrupts junctional protein organization, whereas PlGF blockade restores junction integrity and strengthens the barrier. These effects are mediated by a VEGFR1-dependent cascade involving PKC, Erk1/2, and nitric oxide synthase, which increases nitric oxide production and induces cytoskeletal changes that promote leakage [9,10,12,13]. In diabetic mouse models, repeated intravitreal anti-PlGF antibody administration reduces retinal vascular leakage, normalizes tight junction morphology, and decreases albumin extravasation, with effects comparable to those of aflibercept or VEGFR2-selective inhibition [4,9]. Collectively, these findings indicate that PlGF negatively regulates inner blood–retinal barrier integrity in diabetic eyes [3,9,11,12,13]. Recent studies also link PlGF signaling to endothelial metabolism, particularly the pentose phosphate pathway (PPP) and antioxidant defense mechanisms. In retinal endothelial cells, PlGF reduces the expression and activity of glucose-6-phosphate dehydrogenase, the rate-limiting enzyme of the PPP, and downregulates antioxidant proteins such as peroxiredoxin-6 and heme oxygenase-1. This metabolic reprogramming limits NADPH production and weakens antioxidant defenses, making junctional complexes more susceptible to reactive oxygen species (ROS)-mediated damage. PlGF blockade reverses these effects by increasing PPP activity, restoring antioxidant protein levels, reducing intracellular ROS accumulation, and improving barrier function. Thus, PlGF promotes barrier dysfunction not only through direct VEGFR1 signaling but also by inducing a pro-oxidant metabolic state in retinal microvessels [11,12,13,17] (Figure 2). This metabolic shift may explain why PlGF inhibition can improve barrier function, reduce ROS accumulation, and attenuate inflammation. Therefore, PlGF likely aggravates diabetic retinal disease through integrated vascular, metabolic, and immune mechanisms rather than through angiogenesis alone [9,10,11,24].

### 4.4. Neurodegeneration

The recognition of DR as a neurovascular disease highlights the need to examine the role of PlGF in retinal neuronal health. Recent evidence suggests that PlGF–VEGFR1 signaling promotes chronic microglial activation and neuroinflammation, which may precede overt vascular leakage. In retinal ganglion cells and Müller glia, PlGF signaling increases oxidative stress by suppressing the PPP, rendering neurons more susceptible to apoptosis. Conversely, PlGF neutralization in diabetic models provides neuroprotection, as indicated by the preserved retinal electrophysiological function, including improved scotopic b-wave amplitude on ERG, and reduced glial fibrillary acidic protein expression. These findings suggest that PlGF inhibition may stabilize the neurovascular unit, although longitudinal studies incorporating patient-reported visual outcomes and contrast sensitivity evaluation are needed to confirm these functional benefits [11,25].

Although preclinical studies suggest that PlGF contributes to neuroinflammation and may regulate Müller glial activation and retinal ganglion cell survival, direct evidence that selective PlGF inhibition improves retinal electrophysiological function or produces clinically meaningful visual outcomes remains limited. Future studies incorporating ERG, microperimetry, contrast sensitivity, and patient-reported outcomes are needed to determine whether PlGF-targeted therapies provide neuro-protective benefits beyond vascular stabilization.

### 4.5. Therapeutic Implications and Drug Positioning

Current evidence supports a therapeutic model in which VEGF-A blockade remains the cornerstone for rapidly controlling edema and angiogenic activity, whereas PLGF-VEGFR1 modulation may provide additional benefits in eyes with persistent vascular leakage, inflammation, fibrosis, or incomplete anti-VEGF responses. Dual-ligand inhibition, as demonstrated with aflibercept, provides clinical proof of concept that simultaneous VEGF-A and PlGF blockade may broaden therapeutic efficacy in selected patients. Although the specific contribution of PlGF inhibition to these benefits remains difficult to determine, the disease-selective biology of PlGF supports its potential role in a precision medicine. In this context, PlGF-related biomarkers could help identify patients most likely to benefit from VEGFR1-targeted therapies [4,7,8,22,26,27,28].

### 4.6. Relevance to Clinical Heterogeneity in DR

Clinical heterogeneity remains a challenge in DR management. Some patients respond well to VEGF-A inhibition alone, whereas others show persistent exudation, inflammation, or fibrosis despite repeated treatment. The evidence suggests that this variability may reflect differences in PlGF-driven signaling, especially in eyes with advanced ischemia or chronic disease activity. In this context, PlGF may function as a biomarker of an inflammatory phenotype and a potential additional therapeutic target. A better understanding of PlGF expression patterns in serum, aqueous humor, and the vitreous may support more individualized treatment selection in DR [7,8,19,20,21,22,23,26,27,28].

## 5. Clinical Evidence and Therapeutic Implications

### 5.1. Ocular Fluid Biomarkers

Clinical studies of aqueous and vitreous samples strongly support a role for PlGF in human DR [3,8,19,20,21]. In patients with proliferative DR, vitreous PlGF concentrations are higher in eyes with active neovascularization than in quiescent PDR or non-diabetic controls and correlate with VEGF-A concentrations and disease activity [3,7,19,21]. Notably, prior intravitreal bevacizumab does not substantially alter vitreous PlGF levels, suggesting that VEGF-A-targeted therapy does not fully suppress intraocular PlGF [3,7,19,21].

Aqueous humor studies have found elevated PlGF levels in early non-proliferative DR patients compared with non-diabetic subjects, indicating that PlGF dysregulation accompanies early microvascular dysfunction rather than only end-stage disease [3,20]. More recent studies have extended these findings to serum and vitreous samples, showing that PlGF levels increase with DR severity and correlate with optical coherence tomographic parameters, including macular thickness and volume [3,21].

### 5.2. Growth Factor and Fibrosis Profiles Under Anti-VEGF Therapy

Vitreous analyses before and after intravitreal bevacizumab in PDR patients show that although VEGF-A levels decline substantially after treatment, PlGF and several pro-fibrotic mediators show smaller or inconsistent changes [3,22]. This differential response suggests that VEGF-A-targeted therapy does not fully address PlGF-mediated leakage, inflammation, or fibrosis [3,22]. These findings support strategies that additionally target PlGF or broadly modulate VEGFR1 signaling in advanced DR [3,4,12,13,22].

### 5.3. Preclinical Performance of PlGF Neutralization

In murine models of DR, including streptozotocin-induced diabetes and Akimba mice, PLGF neutralization reduces vascular leakage, leukostasis, neovascularization, and gliosis [3,4,14]. These studies show that anti-PlGF treatment achieves anatomic improvements comparable to those obtained with VEGFR2 blockade or aflibercept, without evidence of retinal neurodegeneration on structural or functional assessment [3,4,14]. Genetic PlGF deletion similarly protects against DR features and increases Akt activation while reducing HIF-1α–VEGF-A signaling [3,14,23].

Notably, selective PlGF inhibition also normalizes endothelial metabolic and redox profiles and strengthens barrier integrity, potentially complementing VEGF-A-targeted therapies [3,11,22,24].

### 5.4. Potential Therapeutic Positioning

Current evidence suggests that modulating PlGF–VEGFR1 signaling may expand the therapeutic landscape of DR. Anti-VEGF-A agents remain indispensable for many patients, but adjunctive or alternative approaches targeting PlGF–VEGFR1 signaling benefit cases with incomplete VEGF responses, prominent inflammation or fibrosis, or concerns about long-term VEGF suppression [3,4,8,12,13,22]. Dual-binding agents such as aflibercept already provide clinical proof of concept that simultaneous VEGF-A and PlGF sequestration can be effective and may offer advantages over VEGF-specific agents in selected subgroup [3,4,5,6,17,18,22,23,26,27,29,30].

PlGF-selective antibodies and other VEGFR1-targeted therapies remain in the early stages of development, and their clinical role will depend on demonstrating additional benefit, acceptable safety, and identifying the patient subsets most likely to benefit [3,4,7,10,12,13]. Biomarker-guided trials incorporating PlGF and VEGFR1-related markers, along with detailed imaging and functional assessments, will be essential to determine whether PlGF-directed interventions can reduce treatment burden and improve long-term visual outcomes in DR [3,8,20,21,22].

Beyond its established roles in angiogenesis and vascular leakage, PlGF may also contribute to the progression of DR by sustaining inflammatory and fibrotic remodeling. In advanced disease, persistent edema and recurrent ischemia often coexist with fibrovascular proliferation and vitreomacular interface abnormalities. PlGF is being increasingly recognized as a key mediator of fibrovascular membrane (FVM) formation, a hallmark of advanced proliferative diabetic retinopathy (PDR). Beyond its angiogenic role, PlGF induces the expression of pro-fibrotic cytokines, including TGF-β and connective tissue growth factor (CTGF), in retinal pigment epithelial (RPE) cells and myofibroblasts. By activating the VEGFR1–PKC–NF-κB axis, PlGF promotes the epithelial–mesenchymal transition (EMT) of RPE cells, facilitating their migration into the vitreous cavity. This pathological remodeling of the extracellular matrix (ECM) causes fibrovascular tissue contraction, resulting in tractional retinal detachment. Unlike VEGF-A, which primarily promotes endothelial cell proliferation, PlGF signaling creates a pro-fibrotic environment that persists even after the initial angiogenic drive is suppressed [3]. These suggests that targeting the PlGF–VEGFR1 axis may be crucial for preventing the long-term scarring and tractional pathology that are often resistant to current anti-VEGF monotherapy.

In this context, PlGF-targeted therapy may offer a more selective approach by attenuating VEGFR1-dependent signaling that promotes leukocyte recruitment, endothelial dysfunction, and pro-fibrotic activity within the retinal microenvironment. PlGF may also have value in a biomarker-guided treatment framework. If intraocular or systemic PlGF levels reflect disease activity, serial measurements could help identify patients with pathology driven predominantly by VEGFR1-mediated inflammation or fibrosis rather than by VEGF-A alone. This information could identify patients likely to respond incompletely to standard anti-VEGF therapy and benefit from adjunctive or alternative PlGF inhibition. Therefore, future clinical studies should incorporate PlGF-related measures alongside anatomical and functional endpoints to determine whether this strategy reduces injection burden and improves long-term visual outcomes in diabetic retinopathy [3,7,20,21,22].

### 5.5. Comparative Analysis: PlGF vs. VEGF Inhibitors in DR Treatment

**Figure 3:**
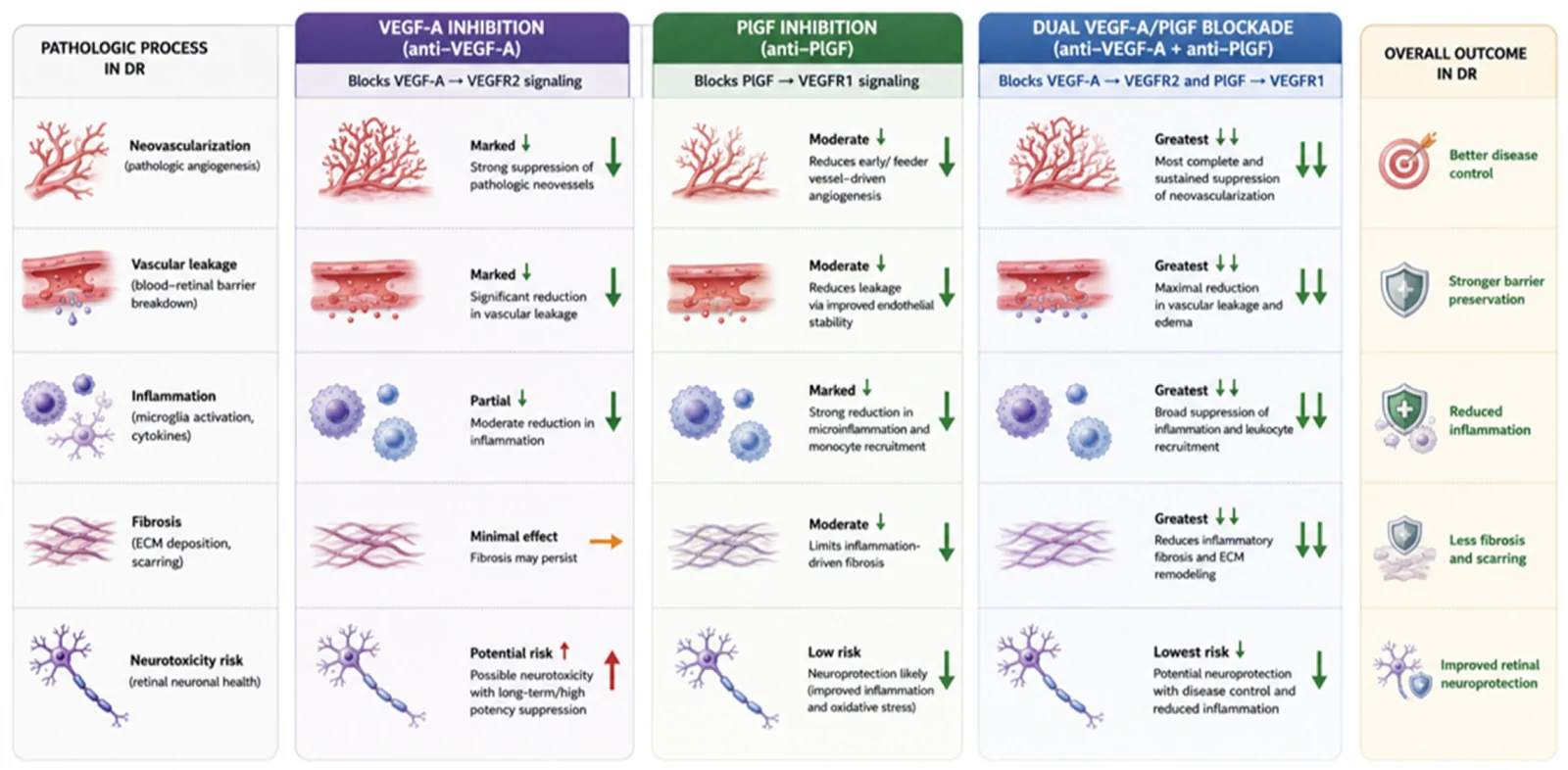
Conceptual/hypothetical model of VEGF-A inhibition, PlGF inhibition, and dual VEGF-A/PlGF blockade in DR. Comparison of their effects on neovascularization, vascular leakage, inflammation, fibrosis, and neurotoxicity risk [3,4,5,6,17,18,22,23,26,27,29,30].

Preclinical head-to-head comparisons in DR models suggest that PlGF inhibition and VEGF inhibition produce overlapping effects on some outcomes but have different effects on others [3,4,14] (Figure 3). Anti-PlGF treatment reduces vascular leakage and neovascularization to a degree similar to VEGF-pathway blockade while exerting stronger effects on microglial activation, leukostasis, and pro-fibrotic markers [3,4,14]. In contrast, VEGF-specific inhibition remains the best-established approach for rapidly suppressing edema and angiogenesis but does not fully inhibit PlGF-mediated inflammatory and metabolic pathways [3,4,8,19,23].

In clinical practice, this distinction is reflected in the pharmacology of available anti-VEGF agents. Bevacizumab and ranibizumab mainly inhibit VEGF-A, whereas aflibercept binds VEGF-A, VEGF-B, and PlGF, functioning as a dual VEGF/PlGF trap. This broader ligand-binding profile may explain the efficacy of aflibercept in some refractory DME cases and its added benefit when PlGF activity is high [3,4,5,6,17,18,22,23,26,27,28,29,30]. However, because aflibercept simultaneously targets VEGF-A, VEGF-B, and PlGF, its clinical benefits cannot be attributed solely to PlGF inhibition, and differences among anti-VEGF agents may also reflect broader pharmacological and pharmacokinetic properties. Therefore, the specific therapeutic contribution of selective PlGF inhibition remains to be established in future clinical trials.

PlGF-directed therapy should also be considered within the rapidly evolving therapeutic landscape for DR, which now includes dual Ang-2/VEGF-A inhibition with faricimab, high-dose aflibercept, sustained-delivery anti-VEGF systems, intravitreal corticosteroids, and combination therapies. While faricimab primarily targets vascular instability through Ang-2 inhibition, PlGF-directed therapy may provide a complementary approach by targeting VEGFR1-mediated inflammation, metabolic dysfunction, and fibrovascular remodeling. In particular, patients with persistent edema, fibrovascular progression, or elevated intraocular PlGF levels could benefit from PlGF-targeted interventions. In this context, selective PlGF inhibition or dual-ligand approaches may be best suited as biomarker-guided adjunctive or second-line therapies for patients who do not achieve satisfactory anatomical or functional responses to current standard-of-care treatments. Overall, the current evidence supports VEGF inhibition as the standard first-line anti-angiogenic strategy, with PlGF-targeted inhibition serving as an attractive complementary strategy for DR characterized by persistent leakage, inflammation, or fibrosis [3,4,8,12,13,14,22].

### 5.6. Biomarker Interpretation and Disease Stratification

A key implication of the PlGF literature is that intraocular and systemic biomarkers may help distinguish vascular, inflammatory, and fibrotic phenotypes in DR. Studies report that aqueous humor and vitreous PlGF levels increase with worsening retinal ischemia and often coincide with elevated VEGF-A and inflammatory mediator levels. This pattern suggests that PlGF is not merely a correlate of disease severity, but is part of a broader molecular signature reflecting active microvascular stress. In practice, PlGF measurements may be most useful when interpreted alongside optical coherence tomography, fluorescein angiography, and clinical markers of persistent edema or recurrent neovascularization [3,7,8,19,20,21,22]. Although biomarker-guided PlGF-targeted therapy is a promoising precision-medicine strategy, several barriers must be overcome before its routine clinical implementation. These include the lack of standardized PlGF assays, validated cutoff values, concordance between systemic and intraocular measurements, minimal invasive sampling limitations, uncertain longitudinal reproducibility, cost-effectiveness concerns, and insufficient prospective validation. Addressing these limitations is essential for reliable implementation of PlGF-guided therapeutic decision-making in clinical practice.

PlGF may help explain incomplete responses to VEGF-A-selective therapy. Despite reduced VEGF-A levels after treatment, persistent inflammation, leakage, or fibrosis, may reflect continued PlGF-VEGFR1 signaling and disease activity. This mechanism is particularly relevant in proliferative DR and refractory diabetic macular edema, where repeated anti-VEGF therapy may not fully normalize the ocular molecular environment. Incorporating PLGF into the biomarker framework could support treatment escalation, earlier switching, or rational combination therapy in selected patients, although prospective clinical validation is required before widespread clinical adoption [7,19,21,22,26,27,28].

### 5.7. Evidence from Dual-Ligand Inhibition

Clinical pharmacology further supports the role of PlGF in therapeutic responses. By binding VEGF-A, VEGF-B, and PlGF, aflibercept has a broader ligand-capture profile than ranibizumab or bevacizumab, which may contribute to improved responses in eyes with more severe disease or prior incomplete treatment responses. Clinical studies in diabetic macular edema indicate that aflibercept provides superior outcomes in eyes with poorer baseline vision, consistent with the potential benefit of broader blockade when PlGF-driven pathology is substantial. Although these findings do not establish a PlGF-specific effect, they align with preclinical studies reporting that selective PlGF inhibition reduces vascular leakage, inflammation, and fibrosis beyond VEGF-A blockade alone. However, since aflibercept simultaneously targets VEGF-A, VEGF-B, and PlGF, its clinical benefits cannot be specifically attributed to PlGF inhibition, and differences among anti-VEGF agents may also reflect broader pharmacological and pharmacokinetic properties. Therefore, the specific therapeutic contribution of selective PlGF inhibition remains to be determined in future randomized clinical trials [22,26,28].

Dual-ligand inhibition does not imply that VEGF-A and PlGF are interchangeable but rather supports partial overlap with distinct biological roles. VEGF-A primarily mediates acute angiogenic and permeability responses, while PlGF contributes more to inflammatory recruitment, barrier dysfunction, and tissue remodeling. This distinction explains why broader inhibitors may benefit selected subgroups while supporting the development of selective PlGF-targeted therapies for phenotype-driven treatment.

### 5.8. Limitations and Future Development

Despite growing evidence, some limitations continue to hinder the clinical translation of PlGF-targeted therapy. First, the evidence for PlGF in DR is largely derived from animal models, particularly streptozotocin (STZ)-induced diabetic mouse and the Akimba model. While these models have provided fundamental insights, they do not fully recapitulate the chronic, systemic, and multifocal nature of human DR. Rodent models, for instance, rarely reproduce advanced fibrovascular proliferation or the long-term neurodegenerative changes observed in human patients. Thus, a translational gap remains as preclinical success with PlGF neutralization has yet to be confirmed in randomized clinical trials (RCTs) specifically evaluating PlGF-selective inhibition [31]. Although most experimental and clinical studies support a pathogenic role for PlGF in DR, the evidence remains heterogeneous. Some studies report variable associations between PlGF levels and disease severity, and the independent contribution of PlGF relative to other angiogenic and inflammatory mediators remains unclear. Furthermore, selective PlGF inhibition has not yet shown clinical efficacy in randomized trials. Furthermore, whether elevated PlGF is a causal driver or a secondary biomarker of ischemic burden remains debated [8]. Second, human biomarker studies are often limited by small sample sizes, cross-sectional designs, and heterogeneous treatment histories, complicating direct comparison across studies. Third, the distribution of PlGF isoforms, receptor availability, and compensatory signaling pathways in the diabetic human retina remain incompletely characterized, especially after chronic anti-VEGF exposure [3,7,8,19,20,21,22]. Although experimental studies strongly support a pathogenic role, persistent PlGF elevation despite vascular stabilization in some clinical observations suggests the existence of complex compensatory pathways [8,32]. Therefore, future studies should determine why some cohorts show limited responses to PlGF blockade, e.g., due to redundant inflammatory pathways or differential VEGFR1 isoform expression. Standardized, reproducible biomarker assays are essential for translating PlGF-targeted therapy from a speculative concept into clinical practice [12]. These unresolved issues highlight the need for well-designed prospective studies to define the precise biological and therapeutic roles of PlGF across the different stages and phenotypes of DR. Additionally, future studies should prioritize longitudinal designs integrating ocular biomarkers, imaging, and functional outcomes. These studies could determine whether PlGF levels increase before clinical deterioration, predict resistance to standard anti-VEGF therapy, and can identify eyes likely to benefit from dual blockade. In parallel, experimental studies should further define PlGF’s interactions with endothelial metabolism, pericyte biology, glial activation, and fibrotic remodeling. The long-term goal is not merely to add another inhibitor but to leverage PlGF biology to refine precision treatment for diabetic retinal disease [3,4,9,10,11,12,13,14,22,23,24,26,27,28].

Beyond its direct vascular effects, PlGF may contribute to diabetic retina pathology by remodeling the inflammatory microenvironment and injury-repair responses. Persistent low-grade inflammation promotes endothelial dysfunction, glial activation, and extracellular matrix remodeling, creating a self-sustaining cycle that drives disease progression. Given its involvement in macrophage recruitment, microglial activation, and pro-fibrotic signaling, PLGF blockade may be particularly beneficial in eyes with recurrent activity despite standard anti-VEGF therapy. This broader biological role suggests that PlGF inhibition could stabilize the retinal environment beyond angiogenic suppression, offering a more comprehensive strategy for complex diabetic retinopathy phenotypes [3,12,14,22,23].

## 6. Conclusions

Accumulating experimental and clinical evidence supports PlGF as a clinically relevant and biologically distinct mediator of DR. In preclinical models, PlGF promotes neovascularization, vascular leakage, inflammatory cell recruitment, reactive gliosis, and fibrovascular remodeling, while its inhibition generally preserves physiological vascular development and does not appear neurotoxic under the studied conditions. Human biomarker studies support this view as they have shown that ocular PlGF levels increase with disease severity, correlate with ischemic and proliferative activity, and remain incompletely suppressed by VEGF-A-directed therapy [7,19,20,21,22].

These findings position PlGF as a potential therapeutic bridge between conventional anti-VEGF monotherapy and broader pathway modulation. While VEGF-A remains the primary first-line target for rapid control of edema and neovascularization, PlGF may address inflammatory, metabolic, and fibrotic disease components that persist after VEGF-A blockade [4,17,18,22,24]. Thus, PlGF inhibition is not a replacement for current treatment but a mechanistically rational complementary approach supported by biological and preclinical evidence. Although selective PlGF inhibition has not yet demonstrated clinical efficacy in randomized clinical trials for DR, and the current human evidence largely derives from observational and biomarker studies, it represents a promising therapeutic approach requiring further clinical investigation. Whether it can reduce treatment burden or improve long-term visual outcomes in selected patients remains to be determined in future prospective clinical trials [4,8,22,26,27,28].

Future progress will depend on biomarker-guided trials, refined disease phenotyping, and direct clinical comparison between VEGF-A-selective and PlGF-inclusive strategies. Studies integrating intraocular PlGF measurements, retinal imaging, and functional outcomes will be critical for identifying patients most likely to benefit from PlGF-directed therapy. Therefore, future studies should incorporate emerging technologies, including OCT and OCT angiography biomarkers, single-cell RNA sequencing, spatial transcriptomics, retinal proteomics, and artificial intelligence-assisted prediction models, to refine disease stratification and improve treatment response prediction. Additionally, the development of combination therapies, gene-based approaches, and sustained drug-delivery systems could expand the clinical applicability of PlGF-targeted interventions within precision ophthalmology. Collectively, the current evidence supports the continued development of PlGF- or VEGFR1-targeted interventions as part of individualized treatment strategies in DR [4,7,8,20,21,22].

## Figures and Tables

**Table 1:** Distinct and overlapping features of VEGF-A and PlGF signaling.

Feature	VEGF-A	PlGF
Major receptor in the retina	VEGFR2 (KDR/Flk-1); also binds VEGFR1	VEGFR1 (FLT1) only
Retinal cell-specific expression	RPE cells, Müller glia, endothelial cells, ganglion cells, pericytes	Müller glia, RPE cells, endothelial cells, infiltrating macrophages/microglia (markedly upregulated under diabetic and ischemic conditions)
Role in normal vasculature	Essential for developmental and maintenance angiogenesis	Limited in physiological angiogenesis
Dominant pathological effects	Angiogenesis, vascular leakage, endothelial proliferation, increased permeability	Vascular leakage, chronic inflammation, leukocyte recruitment, fibrosis, metabolic dysfunction
Major downstream signaling pathways	VEGFR2 → PI3K/Akt, ERK1/2, p38 MAPK, PLCγ/PKC, STAT3, eNOS	VEGFR1 → PKC–ERK1/2–eNOS, NF-κB, JNK/p38 MAPK, ROS generation, pentose phosphate pathway suppression, inflammatory cytokine production
Effect on the blood–retinal barrier	Disrupts tight junctions (ZO-1, occludin, claudin-5) and increases vascular permeability	Potently disrupts barrier integrity through inflammatory signaling; inhibition restores tight junction proteins and vascular stability
Representative therapeutic agents	Ranibizumab, Bevacizumab, Aflibercept, Faricimab (indirect VEGF-A inhibition via VEGF-A binding)	Aflibercept (PlGF trap), Conbercept, OPT-302 (investigational), anti-PlGF monoclonal antibodies (preclinical)
Potential adverse effects of inhibition	Long-term inhibition may impair physiological vascular homeostasis, neuroprotection, wound healing, and choriocapillaris maintenance	Preclinical studies suggest minimal effects on normal retinal vasculature; long-term clinical safety remains under investigation
Level of supporting evidence	Extensive clinical evidence (multiple Phase III trials and real-world studies)	Strong preclinical evidence with emerging clinical evidence; limited Phase II/III validation

## Data Availability

No new data were created or analyzed in this study. Data sharing is not applicable to this article.
